# Framing the world: Strategic narratives of foreign states in China’s mainstream media (1950–2019)

**DOI:** 10.1371/journal.pone.0351772

**Published:** 2026-06-26

**Authors:** Zhicong Chen, Zhengyi Liang, Zhenyu Wang, Xinya Jiang, Cheng-Jun Wang

**Affiliations:** 1 Department of Communications and New Media, National University of Singapore, Singapore, Singapore; 2 Department of Communication, University of California, Davis, California, United States of America; 3 Institute of Journalism Studies, Shanghai Academy of Social Sciences, Shanghai, China; 4 School of Journalism and Media at the University of Texas at Austin, Austin, Texas, United States of America; 5 School of Journalism and Communication, Nanjing University, Nanjing, Jiangsu, China; Universidade Federal do Tocantins, BRAZIL

## Abstract

This study investigates how mainstream Chinese media have portrayed foreign states over the past seven decades. While scholars have extensively studied foreign media portrayals of China, little systematic or longitudinal evidence exists on how China’s official party press frames the world across different countries and time periods. Using word embedding techniques, this study traces media representations of major foreign countries (N = 94) on People’s Daily (1950–2019). Results show that media favorability trends in the People’s Daily closely mirror major turning points in Chinese foreign policy. Regression analyses reveal that a country’s economic development, industrialization, and military capacity are major predictors of its media image, while the influence of economic factors shifted significantly over time. These patterns reflect broader geopolitical transformations and China’s evolving global orientation. The findings illuminate how computational approaches can recover the structural and strategic logics of state media framing, with implications for ongoing debates on narrative power, cultural influence, and China’s evolving role in the global information order.

## Introduction

In contemporary international politics, national images are not passive reflections of “objective” realities. They are socially produced meanings that travel through discourse, diplomacy, and media, shaping how states interpret one another’s intentions, identities, and legitimacy [1–4]. For China, this meaning-making task has become increasingly central to external communication and public diplomacy, where the goal is not only to “be understood,” but to stabilize a preferred interpretive environment for China’s development and international engagement. Media are pivotal in this process because they do more than report events. They provide the vocabulary, associations, and narrative templates through which foreign countries are rendered intelligible to domestic audiences and, indirectly, to the wider world.

This study focuses on People’s Daily, the Chinese Communist Party’s flagship newspaper and a key node in China’s official communication system. As a party organ, People’s Daily functions as an institutional narrator that translates foreign affairs into politically legible stories, reinforces policy-relevant interpretations, and signals shifting diplomatic orientations. These features make it a uniquely valuable corpus for examining how China constructs foreign country images over long historical periods [5,6]. Yet, existing research on international image-building and foreign-country portrayals has two persistent limitations. First, much of the literature emphasizes short windows, specific crises, or single bilateral relationships, which constrains our ability to identify long-run shifts and path dependencies in representations. Second, many studies rely on manual coding and qualitative interpretation alone, limiting scalability, reproducibility, and the detection of subtle semantic changes that accumulate across decades.

To address these gaps, this paper integrates three complementary frameworks. First, the constructivism framework treats international relations as a social structure in which identities and interests are produced through intersubjective meanings. Media discourse is therefore not merely “coverage,” but constitutive practice that helps define who “we” are and who “they” are [1,2,7,8]. Second, the hierarchy of influences model explains how media content is shaped by nested forces, from individual journalists and professional routines to organizational constraints, extra-media actors, and overarching ideological systems [9,10]. This is especially relevant for party media, where ideological and institutional pressures can systematically structure selection, tone, and framing [6,11]. Third, the strategic narrative approach conceptualizes state communication as purposeful storytelling aimed at shaping interpretations of international order, national identity, and specific policy issues. Strategic narratives link domestic legitimacy, foreign policy goals, and communicative power in a fragmented media environment [12,13].

Empirically, we construct a 70-year panel dataset (1950–2019) from People’s Daily and use diachronic word embedding methods to quantify how foreign countries are positioned in the newspaper’s semantic space over time. Word embeddings are well suited for this task because they recover relational meanings from large corpora and can track gradual shifts in associations across historical periods [14,15]. We then link these time-varying representations to changes in China’s bilateral relations and to structural attributes of target states (for example, economic development and military capacity), allowing us to test how material and relational factors jointly shape mediated images. Guided by constructivist international relations theory and strategic narratives scholarship, this study uses computational text analysis of the People’s Daily to advance a longitudinal and mechanism-aware account of China’s official media framing, tracing how foreign country images are constructed, how they respond to diplomatic realignments, and how target-state characteristics condition narrative tone and positioning.

## Literature review

### Constructivism, discourse, and mediated international relations

Constructivist scholarship challenges the assumption that state interests and identities are fixed inputs into international politics. Instead, it argues that identities, interests, and practices are socially constructed through ongoing interaction among actors situated in specific historical, cultural, and ideological contexts [1–4,16]. In this view, the international system is not purely material. It is also ideational. Shared norms, collective identities, and interpretive frames shape how states define situations, evaluate others, and decide what actions are legitimate or appropriate [7,8]. Constructivism therefore directs analytical attention away from “objective” capabilities alone and toward the production of meaning that makes those capabilities politically consequential.

The intellectual roots of this position extend beyond International Relations studies. Interpretive traditions in sociology and social theory foreground meaning-making as central to social order. Classic sociological thinking emphasized how subjective interpretation and legitimacy underwrite institutions and collective life, which later constructivist work adapts to global politics. Post-structuralist scholarship further strengthened this emphasis by treating language and symbols as constitutive of political reality rather than merely descriptive. Discourse theory and deconstruction, for example, highlight how categories, binaries, and labels organize what can be said, what can be believed, and what can be justified [17,18]. This lineage supports a key constructivist claim. International relations can be approached as discursive fields where meaning and power are co-produced through communicative practices.

Within this tradition, discourse is not peripheral. It is one of the main sites where meanings about other states are stabilized, contested, and institutionalized. Including meanings about their character, status, intentions, and moral standing [19,20]. These meanings matter because they feed into how states understand threats and opportunities, how they define interests, and how they justify policy. The constructivist critique of realism and liberalism follows directly. Realism’s material determinism and liberalism’s institutional optimism both underplay how interpretation and identity shape action. Constructivism treats even “anarchy” and “cooperation” as outcomes of shared understandings that evolve through interaction, not as fixed properties of the system [1,2]. The implication is dynamic. International relations are not a stable equilibrium of forces. They are an evolving social process in which identities and relationships transform through sustained communication and repeated interaction [7,21].

Media become analytically central in this account because they scale up discourse. They circulate categories, labels, and evaluative cues that make foreign affairs cognitively legible and morally interpretable. They also help produce socially shared “common sense” about what the international system looks like and where different actors belong within it. This is especially true for official or party media, which often function as authoritative narrators that standardize interpretation and signal preferred readings of international events. For studies of foreign country images, constructivism thus motivates two expectations. First, portrayals of “others” are part of domestic identity construction, because defining foreign states helps define the boundaries and virtues of the national “self.” Second, representation is historically contingent. Shifts in geopolitical relationships, diplomatic alignments, and normative priorities should be observable as shifts in discourse. Including in official media corpora where representations are recorded and reproduced over long periods.

### Strategic narratives as instruments of communicative power

While constructivism clarifies why meaning matters, the strategic narrative framework specifies how political actors attempt to shape meaning in ways that support concrete foreign policy projects. Strategic narratives are coherent stories about international order, national identity, and policy issues that actors project to structure how audiences interpret events and allocate responsibility [12]. Importantly, narrative influence is not equivalent to “having attractive assets.” It is a communicative process in which narratives are produced, circulated, and interpreted within shifting media environments [13]. This approach is widely used to connect public diplomacy and soft power to the practical mechanisms through which influence succeeds or fails, including contestation and reinterpretation by third parties [13,22].

Strategic narratives provide political actors with a means to construct shared interpretations of the past, present, and future of international politics in ways that shape the behavior of both domestic and international audiences [12]. Through these narratives, actors seek to extend influence, manage expectations, and structure the broader discursive environment in which they operate. Strategic narratives encompass stories not only about specific states but also about the international system itself, articulating both collective identity about who “we” are and visions of the desired global order. Their purpose is inherently instrumental: in the short term, they are crafted to guide how others respond to unfolding events, while over the longer term, they can shape how audiences understand their interests, identities, and the trajectory of international relations.

For official media analysis, strategic narrative theory directs attention to at least two narrative layers that can be empirically examined in text. First, system narratives define how the world works (for example, peaceful development versus bloc confrontation). Second, identity narratives define who China is and who foreign states are (partner, competitor, threat, model) Third, issue narratives define what is at stake in specific domains such as security, trade, sovereignty, or development [12]. The relative salience of these narratives may vary across historical contexts. In periods characterized by strong ideological contestation, identity narratives may play a more central role in structuring how foreign states are categorized and evaluated. In contrast, in more pragmatic and internationally integrated periods, system narratives that emphasize development, cooperation, and global order may become more dominant. Accordingly, a foreign state may be portrayed as a partner in one period but as a threat in another. This distinction provides a useful framework for interpreting changes in media portrayals of foreign countries over time.

The hierarchy of influences model further provides a production-side explanation for why media narratives take the form they do. Shoemaker and Reese theorize media content as the outcome of nested constraints and incentives, spanning individual-level factors, newsroom routines, organizational imperatives, extra-media forces (such as state institutions and sources), and macro-level ideology [9,10]. This multi-level architecture is especially useful for studying party media, where ideological and institutional forces are not background conditions but explicit drivers of selection, emphasis, and evaluative tone.

China’s propaganda and media governance system institutionalizes these higher-level influences through formal organizations, policy directives, personnel management, and agenda coordination, creating durable pathways through which foreign policy priorities and legitimacy concerns shape coverage [5,6,11]. At the same time, the hierarchy of influences model cautions against reducing content to ideology alone. Organizational routines (for example, reliance on official sources), professional norms (such as “authoritative” tone), and institutional positioning within the party-state can generate systematic patterns that persist even when immediate diplomatic contexts shift. In this sense, the hierarchy of influences model helps disentangle what is reactive (short-run adjustments to events) from what is structural (stable constraints of the communication system).

### People’s daily as a research subject

People’s Daily offers a uniquely valuable corpus for analyzing the long-run coevolution of ideology, media discourse, and national identity in modern China. As the Chinese Communist Party’s flagship newspaper, it functions not only as a record of international events but also as a major institutional channel for articulating political narratives, legitimizing policy directions, and standardizing how foreign affairs should be interpreted domestically. This dual role. Reflecting diplomatic realities while actively constructing public perceptions. Makes People’s Daily especially suitable for studying how China narrates the external world across changing geopolitical eras.

A growing body of scholarship has used People’s Daily to trace transformations in official discourse over decades, demonstrating that quantitative and computational approaches can recover shifts in framing and narrative priorities at scale. Early work already showed that foreign portrayals in the paper respond sharply to geopolitical realignments. Lee [23], for example, documented dramatic changes in coverage of the United States and the Soviet Union during the 1970s as Sino-U.S. relations normalized. Later, Chang et al. [24] argued that People’s Daily converts news discourse into “social knowledge,” providing citizens with a state-structured interpretive framework for understanding international affairs. More recent studies extend this insight through focused country cases. Research on Japan’s portrayal, for instance, finds alternating positive and negative frames that track diplomatic and territorial events, reinforcing the claim that official media portrayals are politically patterned rather than stylistically incidental. Yet the literature remains constrained in two ways. Empirically, most studies examine single bilateral relationships or short time spans. Methodologically, many rely on qualitative interpretation or manual coding, which limits comparability across countries, scalability across decades, and reproducibility across research teams [25].

Computational text analysis enables the next step. Systematic, multi-country, multi-decade mapping of foreign images with scalable measurement. Word embeddings are particularly useful because they estimate semantic representations from distributional patterns in large corpora, capturing relational meaning rather than only keyword frequency [26–28]. When extended diachronically, embeddings can track how associations shift over time, offering a principled way to operationalize foreign country “images” as changing positions in a semantic space [15,29,30]. This approach has been used to quantify long-term changes in stereotypes and group representations, showing sensitivity to both gradual cultural drift and discontinuities around major historical events [14]. Diachronic embeddings allow us to measure how foreign states are positioned relative to evaluative and thematic dimensions over seven decades, and then relate those shifts to changing bilateral relations and structural attributes such as economic development, industrialization, and militarization. This design directly addresses the central gap in prior work. It moves beyond case-based snapshots toward a comparative, longitudinal, and reproducible account of how China’s official media frames the world. Accordingly, the study is guided by the following research questions:

***RQ1:*** *How did the strategic narratives of foreign states on People’s Daily evolve from 1950 to 2019?*

***RQ2:*** *How do structural attributes, such as economic development and military capability, shape the strategic narratives of foreign states on People’s Daily?*

## Methods

### Data collection

Data for this study were collected from the People’s Daily official database. The collection procedures fully complied with the terms and conditions by the database provider. No personal or sensitive information was accessed, and the data were used solely for academic research purposes in accordance with the permitted use outlined by the source. We preprocessed the corpus in three steps. First, we cleaned the texts to remove obvious non-content noise (for example, duplicated boilerplate and formatting artifacts). Second, we conducted Chinese word segmentation using Python’s Jieba toolkit to transform raw strings into analyzable token sequences. Third, we trained year-specific word embedding models to represent words in a shared high-dimensional semantic space. In embedding space, each word is mapped to a vector, and semantic proximity is operationalized using cosine similarity. This enables us to quantify how a target country is positioned relative to evaluative cues and narrative descriptors in a given year, and to track shifts in these associations over time.

### Measuring media favorability with word embeddings

To capture how the People’s Daily portrays foreign countries over time, we operationalize media favorability using the Skip-Gram with Negative Sampling (SGNS) word embedding model [26]. The word embeddings were trained separately for each year (300 dimensions, context window = 4, subsampling threshold = 1e-5, negative samples = 5, skip-gram, 5 iterations). We then apply the Orthogonal Procrustes algorithm to align yearly embedding spaces, ensuring cross-temporal comparability of cosine similarities. Semantic orientation scores are computed using WEFAT (Word Embedding Factual Association Test), which quantifies each target country’s relative association with the friend and enemy word lists. Specifically, we defined set A to include terms associated with hostility (e.g., “enemy,” “foe”) and set B to include terms linked to friendliness (e.g., “friend,” “ally”). Given a particular country, such as the United States, we calculated the relative score on the enemy-friend spectrum, thereby quantifying People’s Daily’s portrayal of that country’s favorability.

The friend/enemy dimension has deep theoretical roots in both Chinese political thought and Western political theory. Mao [31] identified the distinction between enemies and friends as the fundamental question of revolution, a principle that has since shaped the ideological framing of China’s foreign relations since the founding of the People’s Republic. This aligns with Schmitt’s [32] concept of the political, which defines political life as structured around the irreducible opposition between friend and enemy. Together, these theoretical traditions suggest that the friend/enemy binary functions as a durable organizing principle of political discourse, supporting the conceptual stability of this dimension across the study’s 70-year observation window. At the operational level, cross-period comparability is further ensured through the Orthogonal Procrustes alignment, which rotates yearly embedding spaces into a shared reference frame without distorting their internal geometry.

### National-level variables

To model external correlates of media portrayals, we compiled country-year covariates from the 2025 release of Cross-National Time Series (CNTS) database [33]. We focus on structural attributes commonly theorized to shape international perceptions and narrative positioning: economic development (national income per capita, log-transformed to address right-skew), industrialization (industrial activity as a percentage of GDP), military size (military personnel as a percentage of the total population), and regime militarization (a measure of the militarization of governance).

### Analytical framework

We construct a country-year panel spanning 1950–2019 by merging embedding-based country favorability scores derived from the People’s Daily corpus with country-level structural indicators from the 2025 CNTS dataset. Country inclusion was determined by data availability rather than substantive preselection, and the full panel comprises 6,419 observations across 94 countries. The dependent variable is the embedding-based favorability score for each country-year; the main independent variables are structural country attributes drawn from CNTS, including measures of economic development and the political and military controls described above.

Given that observations are repeatedly nested within countries, we estimate linear mixed-effects models with country-level random intercepts to account for unobserved time-invariant heterogeneity in baseline portrayals, alongside fixed effects for the substantive predictors. This specification allows us to decompose within-country temporal variation from between-country differences in average positioning. To assess the model specifications, we additionally estimated two-way fixed-effects and two-way random-effects models using the same covariates. Estimates were highly consistent across all three specifications, and a Hausman test comparing the fixed- and random-effects estimators yielded a non-significant result (p = 0.8567), confirming that the random-effects assumption is not violated. While two-way fixed-effects models are well-suited to isolating within-country variation, they remove all between-country differences and absorb common time-specific heterogeneity, which are substantively central to our research questions. Because our analytical interest lies in both within-country temporal change and systematic cross-national variation, we retain the mixed-effects specification with random intercepts as the primary analytical framework.

## Results

**Fig 1:**
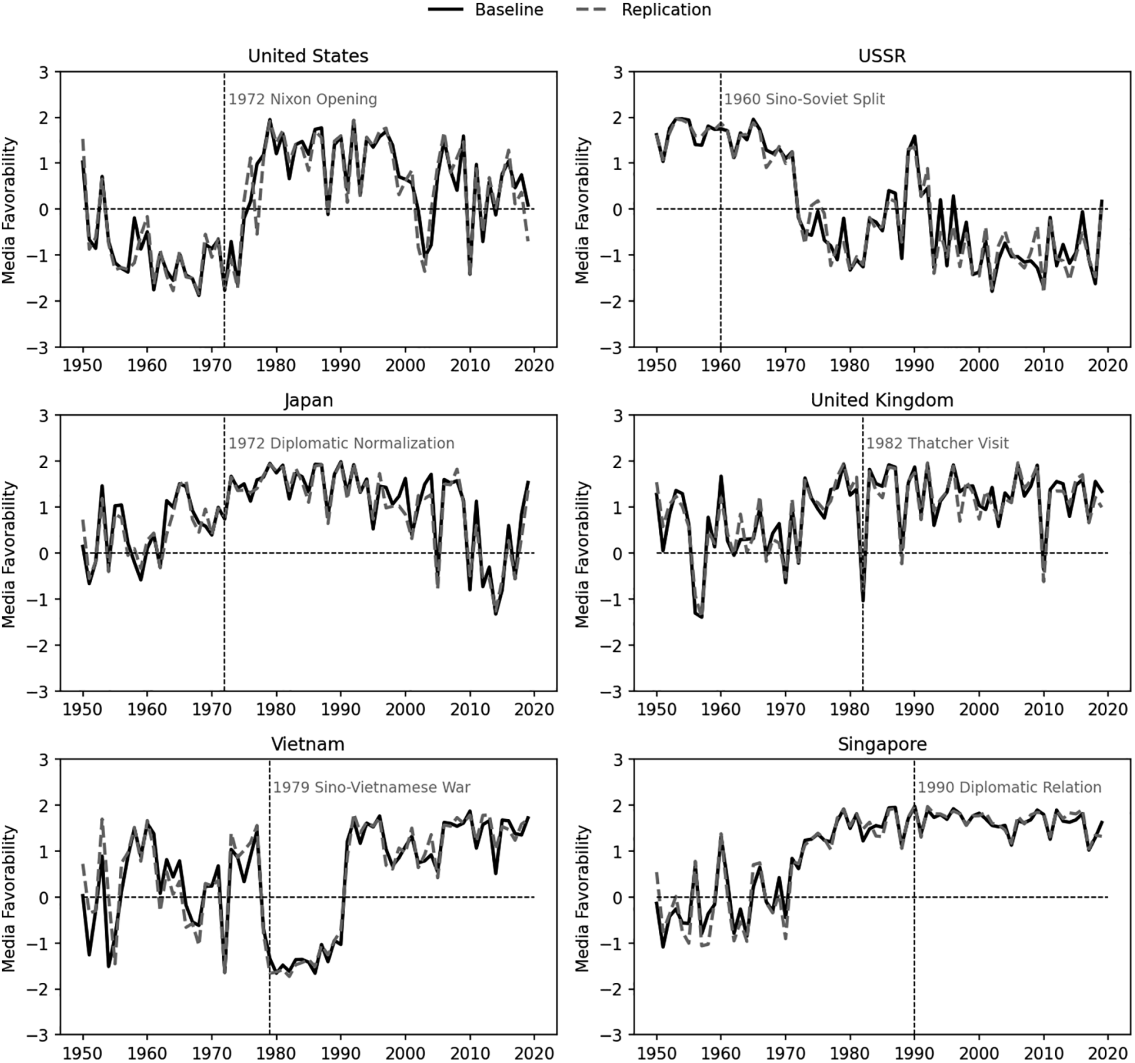
Cross-model comparison of media favorability toward six countries.

We begin by examining longitudinal changes in the People’s Daily’s portrayals of major foreign countries across the study period. Fig 1 compares favorability trends toward the United States, the Soviet Union, the United Kingdom, Japan, Vietnam, and Singapore across embedding models. To assess robustness to modeling parameters, we trained an independent second SGNS model under identical hyperparameter settings (the replication model) to evaluate the stability of the embeddings across stochastic training runs, ensuring that the observed semantic structures are not artifacts of a single training instance. The overall temporal patterns are consistent across the baseline and replication models, suggesting that the substantive findings are not driven by a particular embedding specification.

Media portrayals are likely correlated with historical events in China’s foreign relations. For the United States and the Soviet Union, the two series capture the geopolitical realignment associated with the Sino-Soviet split and the opening to the United States in the 1970s: portrayals of the Soviet Union become increasingly negative, while depictions of the United States grow more favorable. This pattern is consistent with prior historical accounts of rhetorical realignment in Chinese official discourse. The United Kingdom and Japan show similar dynamics. Following the normalization of relations in 1972, portrayals of both countries become more favorable overall, although each series also reflects periods of instability tied to specific political events, such as the 1967 Hong Kong riots in the British case and later territorial tensions in the Japanese case. Vietnam follows a contrasting trajectory: favorability drops sharply in the late 1970s during the period of military confrontation, before recovering after the end of the Cold War and the improvement of bilateral relations. Singapore also displays a notable long-run shift. Early portrayals are relatively negative, reflecting its association with Western-aligned regional politics, but this pattern moderates over time, and favorability rises substantially after the expansion of bilateral ties in the reform era and the establishment of formal diplomatic relations in 1990.

We then turn to the multivariate analysis to examine the structural predictors of cross-national variation in favorability. As country coverage varies across country-years in both the text-derived measures and some covariates, we conduct all analyses on two samples in parallel: the full 94-country panel and a balanced sample restricted to the 65 countries with complete data coverage throughout the entire 70-year period. Table 1 presents linear mixed-effects models for the full and balanced samples. Models 1–3 use the full sample, while Models 4–6 use the balanced sample, which is restricted to countries with complete coverage of the focal outcome across all years from 1950 to 2019. In the full sample, economic development is positively and significantly associated with media favorability across all specifications, with coefficients declining from B = 0.217 in Model 1 (p < .001) to B = 0.122 in Model 3 (p < .001) after the inclusion of additional controls and the interaction term. In the balanced sample, the same positive pattern remains, with coefficients ranging from B = 0.248 in Model 4 (p < .001) to B = 0.177 in Model 6 (p < .001). Political system is negatively associated with favorability in the controlled models (B = −0.086, p < .01 in the full sample; B = −0.075, p < .05 in the balanced sample), and military size also shows a small but consistently negative association (p < .001). Industrialization is positive but less consistent, reaching significance only in the most fully specified models. The interaction between economic development and year is positive and statistically significant in the final model for both the full sample (B = 0.002, p < .001) and the balanced sample (B = 0.002, p < .05), suggesting that the association between economic development and favorability becomes stronger over time. For the balanced-sample analyses, we restricted the data to countries with complete coverage on the focal outcome across the full 1950–2019 period.

**Table 1:** Mixed-effects models for SGNS 2026 on the core outcome across full and balanced samples.

	SGNS 2026 Full			SGNS 2026 Balanced		
Variable	Model 1	Model 2	Model 3	Model 4	Model 5	Model 6
Intercept	0.552***	0.708***	0.759***	0.584***	0.685***	0.723***
	(0.055)	(0.085)	(0.086)	(0.067)	(0.104)	(0.106)
Economics	0.217***	0.203***	0.122***	0.248***	0.238***	0.177***
	(0.017)	(0.019)	(0.030)	(0.021)	(0.022)	(0.035)
Year	−0.004***	−0.004***	−0.007***	−0.005***	−0.005***	−0.008***
	(0.001)	(0.001)	(0.001)	(0.001)	(0.001)	(0.002)
Industrialization		0.002	0.004*		0.003	0.004*
		(0.002)	(0.002)		(0.002)	(0.002)
Political System		−0.086**	−0.094**		−0.075*	−0.081*
		(0.029)	(0.029)		(0.033)	(0.033)
Military Size		−0.001***	−0.001***		−0.001***	−0.001***
		(0.000)	(0.000)		(0.000)	(0.000)
Economics × Year			0.002***			0.002*
			(0.001)			(0.001)
Random Effects	country	country	country	country	country	country
σ²	0.635	0.596	0.594	0.648	0.605	0.603
τ₀₀	0.226	0.214	0.220	0.238	0.246	0.254
ICC	0.262	0.264	0.270	0.269	0.289	0.296
N	93	90	90	65	64	64
Observations	5,357	4,387	4,387	3,876	3,236	3,236
Marginal R²/ Conditional R²	0.056/ 0.304	0.070/ 0.315	0.079/ 0.328	0.070/ 0.320	0.091/ 0.354	0.099/ 0.366

Note: *p < 0.05 **p < 0.01 ***p < 0.001.

**Fig 2:**
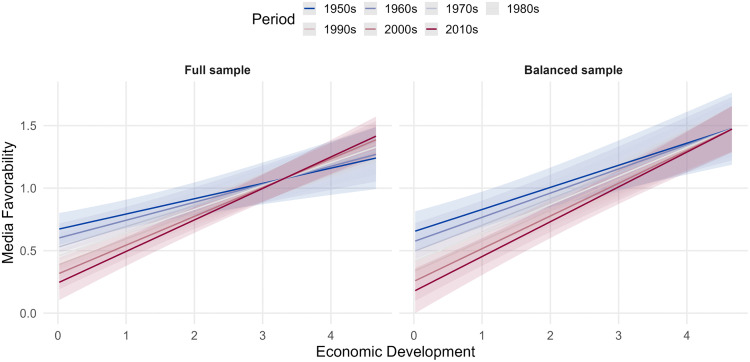
The association between economic development and media favorability every ten years.

Further analysis indicates that the association between national income level (Economic Development) and media favorability is historically contingent, as reflected in the positive interaction between economic development and year in the fully specified model. Fig 2 shows that during the 1950s, 1960s, and 1970s, the marginal relationship between economic development and favorability is generally flat or negative, indicating that more affluent countries were not consistently depicted in more favorable terms. From the 1980s onward, however, the slope becomes positive and grows progressively steeper across subsequent decades. This pattern suggests that the symbolic meaning of economic development in Chinese state-media portrayals shifted over time, becoming increasingly aligned with positive evaluations in the post-reform period.

**Fig 3:**
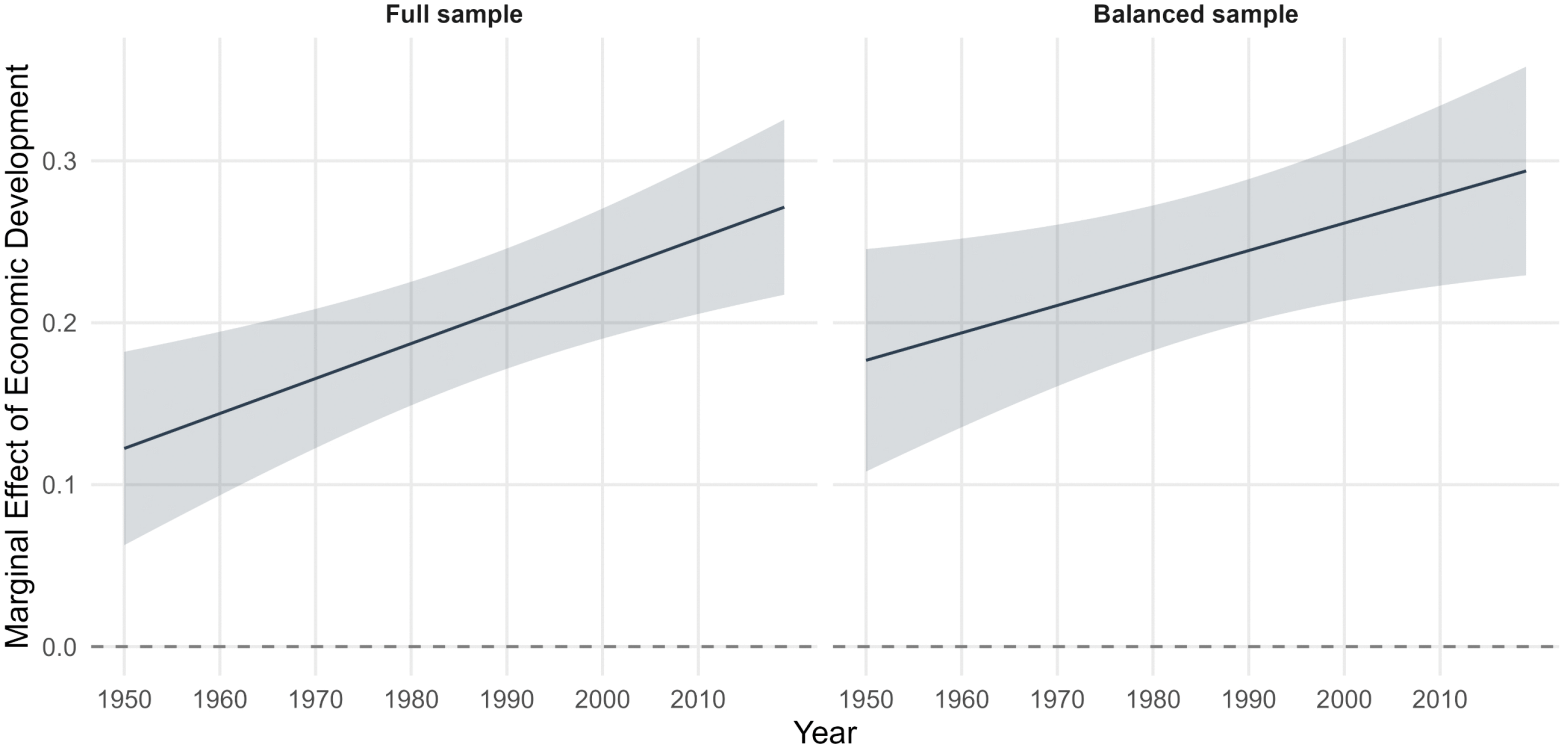
Marginal effect of economic development in regressions over time.

Fig 3 traces the marginal effect of national income level on media favorability across the full study period. In the early decades, especially from the 1950s through the 1970s, the marginal effect of economic development was negative, indicating that higher-income countries were associated with less favorable portrayals in the People’s Daily. This negative relationship diminishes over time and appears to reverse from the reform era onward. By the 1990s and continuing through the 2000s and 2010s, the marginal effect is positive, suggesting that wealthier countries were increasingly portrayed in more favorable terms.

## Discussion

From 1950 to 2019, People’s Daily exhibits a sustained long-run shift toward more moderate and friendly portrayals of foreign countries. Beneath this aggregate trend, however, lies considerable temporal variation: individual country depictions fluctuate across periods, reflecting the episodic rhythms of bilateral relations, geopolitical realignments, and specific historical events. From a constructivist lens, both the long-run trajectory and these shorter-run fluctuations are not simply “sentiment drift.” They reflect a durable and ongoing process of meaning-making in which foreign states are continuously repositioned within China’s evolving understanding of world order, threats, partners, and development opportunities [1,2].

The second core finding is that a country’s economic development is positively associated with favorability, and that association strengthens over time, especially after China’s reform era. Substantively, this implies that “economic capability” becomes an increasingly salient cue in the newspaper’s representation of foreign states. In the strategic narrative framework, this is consistent with a shift in the dominant issue narratives. From revolutionary struggle and security-centric storylines toward development, modernization, and cooperation as the primary interpretive template for external relations. Strategic narratives work by projecting coherent stories about the international system, the actor’s identity, and specific issues, then inviting audiences to interpret events through that narrative structure [12, 13]

A third pattern is that structural attributes beyond economics, including militarization, correlate with portrayals. The point is not that People’s Daily mechanically “rewards” wealth or “penalizes” military power. Rather, it suggests that external structural signals are selectively translated into discourse, as the newspaper encodes what kinds of states are legible as partners, rivals, threats, or exemplary models under China’s prevailing worldview in a given period. This is where a hierarchy of influence models becomes useful. It explains how macro conditions become media content through layered filters, from the social system and institutional environment down to organizational priorities and routine production practices [9,10].

### Theoretical and methodological implications

Importantly, the interpretation of these findings should be situated in the evolving institutional role of People’s Daily itself. Across the period from 1950 to 2019, the newspaper has shifted from a highly ideological party organ during the Maoist era, to a more journalistic and outward-facing publication during the reform period, and more recently to a central channel for ideological guidance [34,35]. These institutional transformations may shape not only the tone but also the underlying logic through which foreign states are represented. For example, the meaning of positivity itself changes as the institutional role shifts. Positive portrayals in Maoist era often reflected ideological alignment, whereas in the reform period, they increasingly signaled economic partnership or diplomatic pragmatism. Recently, positivity has represented strategic narrative management and global image projection.

Similarly, the growing salience of economic development in predicting favorability likely reflects not only changes in the global system, but also a shift in the newspaper’s evaluative frameworks toward modernization and development narratives. These patterns are consistent with strategic narrative theory [12], as the increasing emphasis on economic development aligns with a broader shift toward development- and cooperation-oriented narratives. As such, the observed long-term trends should be understood not as uniform sentiment shifts, but as the product of historically contingent media practices embedded in changing political and institutional contexts.

The findings contribute to constructivism by showing that state identity and the construction of “others” can be observed directly in a central media corpus, rather than inferred from episodic policy statements or elite interviews. Constructivism holds that international meanings and role identities are produced through social practice and shared interpretation [1,2,7,8]. This study extends that claim empirically by demonstrating that the People’s Daily functions as a durable discursive institution that consistently stabilizes categories of friendship, threat, and partnership across decades. The long-run shift toward more positive portrayals is therefore not merely stylistic moderation: it reflects a measurable reconfiguration of the interpretive frames through which foreign states are made legible to domestic audiences and through which China positions itself within a changing international order.

Building on this constructivist foundation, the findings also advance strategic narrative theory by specifying how these interpretive frames not only organize meaning but actively shape how audiences make sense of their interests, identities, and the broader international order. Strategic narrative research emphasizes that actors project system, identity, and issue narratives to structure how audiences interpret world order and policy choices [12,13]. This trajectory suggests a gradual reorientation in how foreign states are positioned within China’s narrative of international order, which may encourage audiences to view external actors less through rigid ideological opposition and more through relational and pragmatic lenses. The strengthening association between economic development and favorability further points to the consolidation of a development-centered system narrative, in which stability and prosperity serve as the primary criteria for evaluating other states. This narrative does not merely describe the world; it helps define what kinds of partners are desirable and what forms of engagement are legitimate.

The significant association between structural attributes, such as militarization, and favorability further suggests that these attributes are incorporated into narrative frameworks that differentiate between partners, competitors, and potential threats, translating structural conditions into meaningful categories that guide audience interpretation. Over time, such patterned representations can shape how domestic and international audiences understand their own positions, alignments, and expectations within the evolving international order. To explain how these meanings are produced consistently at scale, the hierarchy of influences framework supplies the missing mechanism linking macro context to media text [9,10,36]. The long time span reveals both stability in the institutional pipeline of authoritative interpretation and change in narrative emphasis: in China’s media system, propaganda institutions and the political economy of media control standardize acceptable interpretations, manage ambiguity, and preserve ideological consistency even under commercialization [5,6,11,37,38]. Taken together, the three frameworks form a single integrated account — constructivism explains why meanings and identities are central; strategic narrative theory specifies the content and evaluative logic of meaning-making; and the hierarchy of influences explains how that logic is routinized and reproduced through institutional media structures.

Methodologically, the study shows how computational text analysis can operationalize these theoretical claims in a historically sensitive way. Diachronic word embeddings support a text-as-data approach that is now central in computational social science and digital humanities [39,40]. Embeddings quantify relational meaning. They capture how countries are positioned within semantic neighborhoods, which fits constructivist and narrative theories that treat meaning as relational, contested, and historically contingent rather than fixed [26,41]. Substantively, this makes it possible to move from illustrative examples of framing to comparative measurement across countries and decades. At the same time, diachronic embeddings require explicit validation because corpus composition, frequency shifts, and alignment choices can generate artifacts that mimic “semantic change” [15,42,43]. The practical implication is straightforward. Computational scaling is only persuasive when paired with robustness checks and theory-driven interpretation that makes clear what the measures capture, and what they cannot claim.

### Limitations and future directions

Several limitations should be noted. First, causal inference remains unresolved. The relationship between national characteristics and media portrayals is correlational, and media representations may also be influenced by other factors, such as diplomatic relations and major geopolitical events. Specifically, the association between economic development and favorability in People’s Daily does not identify directionality or mechanism. Economic ties may shape discourse, discourse may facilitate economic strategy, or both may be driven by third factors such as diplomatic normalization, trade dependence, or security alignments. Second, scope and measurement constrain generalizability. Focusing on a single outlet improves internal coherence but centers an unusually authoritative “official voice,” which may not represent the broader Chinese media ecology. In addition, favorability captures only one slice of foreign image. Strategic narratives can be positive yet paternalistic, cooperative yet distrustful, or respectful yet threat-oriented. Third, word embeddings are sensitive to preprocessing, corpus composition, and alignment choices, and can reproduce biases in language data [42,44]. It should also be noted that while Orthogonal Procrustes alignment improves the geometric comparability of embedding spaces trained on different corpora, it does not fully resolve deeper contextual semantic drift across radically different political and linguistic eras. Therefore, cross-period comparisons should be interpreted as capturing broad directional trends rather than precise semantic equivalences. Finally, the analysis focuses on encoded representations rather than reception, so it cannot directly address how audiences decode, contest, and circulate these portrayals [45].

Future research can address these issues with more targeted designs and broader data. To strengthen causal leverage, event-centered and quasi-experimental approaches that exploit discrete geopolitical shocks would be more defensible than correlational inference, including interrupted time series around major diplomatic turning points and synthetic control analyses that build counterfactual trajectories for key bilateral relationships [46,47]. To improve generalizability and better operationalize the hierarchy of influences, comparative analyses across Xinhua, CCTV, provincial party papers, and market-oriented outlets could test whether similar narrative logics hold under different organizational constraints [9,37,38]. Measurement should also move beyond a single favorability axis toward multidimensional representations, combining embeddings with supervised stance or sentiment models and topic models that identify the issue domains driving evaluation [48]. Finally, linking People’s Daily narratives to downstream uptake, through republishing networks, policy documents, foreign media citations, or audience discussions, would allow strategic narrative effects to be examined as an empirical process rather than assumed.

## Conclusions

Overall, this study shows that China’s official portrayals of foreign states are produced through an integrated process linking meaning, narrative strategy, and institutionalized media production. From a constructivist perspective, People’s Daily helps constitute international meanings by repeatedly defining who foreign states are. Partners, rivals, threats, or models. And by situating China’s own identity within an evolving world order. From a strategic narrative perspective, these portrayals function as long-run storytelling that organizes international affairs around preferred interpretations, especially a growing emphasis on development and cooperation after the reform era. From the hierarchy of influences perspective, these meanings and narratives are not simply the choices of individual writers. They are stabilized and reproduced through layered constraints, from the party-state’s ideological and institutional environment to organizational routines of authoritative reporting. Empirically, the 1950–2019 diachronic embedding evidence matches this integrated account. Foreign images trend to be more positive over time, and their favorability systematically tracks structural features of target states, with economic development becoming increasingly predictive. Together, the three lenses explain why People’s Daily operates simultaneously as a mirror of China’s external relations and a mechanism for narrating the world in ways that align geopolitical context, development priorities, and institutional communication power.
